# 淋巴管生成与非小细胞肺癌淋巴转移的机制研究进展

**DOI:** 10.3779/j.issn.1009-3419.2021.101.42

**Published:** 2021-12-20

**Authors:** 夏尧 刁, 超 郭, 单青 李

**Affiliations:** 100730 北京，中国医学科学院，北京协和医学院，北京协和医院胸外科 Department of Thoracic Surgery, Peking Union Medical College Hospital, Chinese Academy of Medical Sciences and Peking Union Medical College, Beijing 100730, China

**Keywords:** 肺肿瘤, 淋巴转移, 淋巴管生成, 肿瘤微环境, 非编码RNA, Lung neoplasms, Lymphatic metastasis, Lymphangiogenesis, Tumor microenvironment, Non-coding RNA

## Abstract

肺癌在我国的发病率和死亡率均排名第一，肿瘤发生转移往往预示患者预后不良。其中，非小细胞肺癌（non-small cell lung cancer, NSCLC）的淋巴转移是导致患者预后不良的重要因素。淋巴管生成是促进肿瘤淋巴转移的重要环节。本文综述肿瘤相关淋巴管生成的分子机制，探讨肿瘤微环境与淋巴管内皮细胞间的相互作用，并总结了目前关于非编码RNA对于肿瘤相关淋巴管生成调控机制的研究及进展，以期为NSCLC相关淋巴管生成的研究与诊疗提供新的思路。

## Abstract (en)

Lung cancer ranks the first cancer-related morbidity and mortality in China. Tumor metastasis always predicts the poor prognosis for patients. Moreover, lymphatic metastasis is one of the most significant predictors of poor prognosis in patients with non-small cell lung cancer (NSCLC) and lymphangiogenesis represents the bridge that functionally facilitates tumor lymphatic metastasis. In this review, we first discussed the molecular mechanisms of tumor-associated lymphangiogenesis and the interaction between tumor microenvironment and lymphatic endothelial cells, then, summarized the role of non-coding RNA in regulating tumor-associated lymphangiogenesis in recent frontier studies, with the aim to provide some novel insights on NSCLC-related lymphangiogenesis research, diagnosis and treatment.

非小细胞肺癌（non-small cell lung cancer, NSCLC）占所有肺癌病例的85%，NSCLC主要包括三种组织病理学类型：腺癌、鳞状细胞癌和大细胞癌^[1, 2]^。尽管医疗技术的发展一定程度上改善了肺癌患者的预后，但NSCLC仍是全球癌症死亡的主要原因之一^[3]^。淋巴转移（lymphatic metastasis）是NSCLC常见的转移途径和独立预后因素，NSCLC术后病理判定的淋巴转移情况已被证明是决定患者长期预后的最重要因素之一，甚至出现淋巴微转移也可预测患者预后不良^[4-6]^。在过去，淋巴管系统常被认为在肿瘤转移中起被动的作用，但越来越多的研究显示，淋巴管在其中发生了主动的改变以促进肿瘤转移，这其中就包括淋巴管生成（lymphangiogenesis）的过程。淋巴管生成指由肿瘤内和肿瘤周围原有的淋巴管进一步新生出更多淋巴管以促进了肿瘤淋巴转移的过程，从而导致患者的预后不良^[7]^。淋巴管生成需要淋巴管内皮细胞（lymphatic endothelial cells, LECs）一系列生物学行为的相互协调，包括增殖、发芽、迁移和成管，这些事件与血管生成的过程相类似^[8]^。近年来，随着对分子生物学研究技术的不断进展，对肿瘤淋巴管生成的分子机制以及NSCLC的淋巴转移机制研究也逐渐深入。现将其目前的研究现状作一综述。

## 1 肿瘤相关淋巴管生成的分子机制

### 1.1 血管内皮生长因子家族（vascular endothelial growth factors, VEGFs）

在肿瘤转移的过程中，肿瘤微环境中存在多种促淋巴管生成因子，促进LECs的增殖和形态改变。其中VEGF-C-VEGFR-3和VEGF-D-VEGFR-3轴被认为是肿瘤相关淋巴管生成的主要驱动因素^[9]^，其中又以VEGF-C促进LECs生长的效果最为显著^[10]^。VEGF-C/D通过与LECs上的受体酪氨酸激酶VEGFR-3结合，激活蛋白激酶C依赖的细胞外调节蛋白激酶（extracellular regulated protein kinases, ERK）1或ERK2通路级联以及蛋白激酶B（protein kinase B, PKB/AKT）的磷酸化，然后其下游通路会进一步介导LECs的生长及迁移^[11, 12]^。VEGF-C/D对LECs的这一调控过程与VEGF-A通过结合VEGFR-2对血管生成进行分子调控的过程相似^[13]^。

除VEGF-C/D-VEGFR-3轴自身外，许多分子也可通过调控此通路在肿瘤相关淋巴管生成中发挥重要作用。例如，环氧合酶2（cyclooxygenase 2, COX2）可以通过催化合成前列腺素以促进VEGF-C的生成，进而促进肺腺癌相关的淋巴管生成和淋巴转移^[14]^。相似的是，前列腺素脱氢酶（prostaglandin dehydrogenase, PGDH）也可以通过调控前列腺素的合成以促进VEGF-D介导的淋巴管生成^[15]^。此外，胶原蛋白和钙结合表皮生长因子结构域-1（collagen and calcium-binding EGF domain-1, CCBE-1）可以增强VEGF-C的蛋白水解以促进LECs的成管及迁移并促进肿瘤淋巴管生成^[16]^。VEGF-C/D除了可与VEGFR-3结合，神经纤毛蛋白-2（neuropilin-2, NRP-2）也可以作为VEGFR-3的共同受体或VEGF-C/D的独立受体介导信号通路以诱导LECs生长、迁移、重建和淋巴管生成^[17, 18]^。由此可见，许多淋巴管生成相关的分子及通路都是围绕VEGF-C/D-VEGFR-3通路进行调控的。

另一方面，一些NSCLC的预后分析也同样证明了VEGF家族在NSCLC中调控淋巴管生成及淋巴转移的重要意义。首先，VEGF-C的表达量与NSCLC的淋巴管密度（lymphatic vessel density, LVD）、淋巴转移和患者预后相关^[19, 20]^。此外，一项研究指出白介素（interleukin, IL）-7/白介素7受体（IL-7 receptor, IL-7R）介导的VEGF-D上调与NSCLC的LVD和不良预后密切相关^[21]^。

### 1.2 其他促淋巴管生成因子

目前除了VEGF-C/D外，还发现了许多其他的促淋巴管生成因子，包括成纤维细胞生长因子（fibroblast growth factor, FGF）^[22, 23]^、表皮生长因子（epidermal growth factor, EGF）^[24]^、血管生成素（angiopoietins, ANGPTs）^[25]^、肾上腺髓质素（adrenomedullin, AM）^[26, 27]^、1-磷酸鞘氨醇（sphingosine-1-phosphate, S1P）^[28]^和促红细胞生成素（erythropoietin, EPO）^[29]^等。例如：FGF-2可与VEGF-C发挥协同作用，通过与FGFR-1结合作用于LECs，从而促进淋巴管生成。然而，这种效应仅出现在VEGFR-3激活的LECs^[23]^，有研究^[30]^显示FGF-2与VEGFR-3在NSCLC中共表达预示患者预后不良。相似的是，EGF与黑色素瘤中的VEGF-C和同源盒基因转录因子1（prospero homeobox protein 1, Prox-1）表达相关，肿瘤来源的EGF与VEGF-C协同作用以促进LECs的生长^[24]^。Holopainen等^[25]^通过对NSCLC荷瘤小鼠的动物实验发现ANGPT2阻断抗体可以减少淋巴结和肺转移，并抑制肿瘤相关淋巴管生成。此外，AM可直接激活ERK下游信号通路以促进LECs生长^[27]^。Nagahashi等^[28]^证实了鞘氨醇激酶1的产物S1P介导了肿瘤相关的淋巴管生成，而且这一过程不依赖于VEGF-A或VEGF-C。Lee等^[29]^的研究表明EPO既可以通过ERK1/2-和PI3K-依赖通路直接诱导LECs的增殖、迁移和成管，也可以通过促进巨噬细胞产生VEGF-C以促进淋巴转移。由此可见，一些分子可以不依赖于VEGF-C/D的信号通路，直接发挥促进淋巴管生成及淋巴转移的作用。而且许多促淋巴管生成因子的作用及调控机制还未在NSCLC中进行验证，仍需要进一步研究去探索及证实。

### 1.3 淋巴管生成抑制因子

目前已经确定了许多促淋巴管生成因子，但其实也存在一些淋巴管生成抑制因子。例如，转化生长因子-β（transforming growth factor-β, TGF-β）在LECs中转导信号并抑制它们的增殖、迁移和成管，并且还可以抑制LECs的标志物[淋巴管内皮细胞透明质酸受体1（lymphatic vessel endothelial hyaluronic acid receptor 1, LYVE-1）和Prox-1]的表达^[31]^。此外，骨形态发生蛋白9（bone morphogenetic protein 9, BMP-9）属于TGF-β超家族，可以作用于其受体的激活素受体样激酶1（activin receptor-like kinase, ALK-1）从而下调Prox-1和细胞周期蛋白的表达，进而抑制LECs的增殖，并且导致了LECs向血管内皮细胞的去分化作用^[32]^。因此，促淋巴管生成因子和淋巴管生成抑制因子可能共同决定了LECs的生物学行为。当两者之间的作用出现失衡时，可能会使机体出现病理状态^[33]^。

## 2 肿瘤微环境与LECs的相互作用

### 2.1 肿瘤细胞与LECs的相互作用

肿瘤细胞本质是追求持续地增殖，因此生长的压力迫使其在肿瘤微环境中不断寻求有利于自身生存和生长的条件，其中就包括LECs^[34]^。多项研究证实，肿瘤细胞不仅是淋巴管生成的关键促进因子VEGF-C/D的重要来源^[35, 36]^，也高表达多种其他的促淋巴管生成因子，例如ANGPT2、FGF-2和血小板衍生生长因子BB（platelet-derived growth factor-BB, PDGF-BB）^[23, 25, 37]^。其中，PDGF-BB也被证实是一种介导淋巴管生成的因子^[37]^，并且PDGF-BB和VEGF-C共表达与NSCLC的淋巴管生成和患者预后不良相关^[38]^。有许多研究揭示了趋化因子与趋化因子受体在肿瘤相关淋巴管生成中的作用，尤其是在肿瘤诱导LECs迁移中的作用。例如：肿瘤细胞来源的CC趋化因子配体（CC-chemokine ligand, CCL）27/28可以与CC趋化因子受体（CC-chemokine receptor, CCR）10结合，从而促进LECs的募集^[39]^。肿瘤细胞可以产生各种因子作用于相应的LECs受体，进而诱导LECs的增殖、迁移以及成管。相反的，一些研究发现LECs也可以向肿瘤细胞发出迁移信号。Shields等^[40]^发现LECs来源的CCL21可以与肿瘤细胞表达的CCR7相结合，从而促进肿瘤细胞迁移。相似的是，当淋巴结的LECs受到局部的炎症因子刺激，可产生CCL1与CCR8相结合，使肿瘤细胞向淋巴结迁移^[41]^。

### 2.2 肿瘤相关成纤维细胞（cancer-associated fibroblasts, CAFs）与LECs的相互作用

CAFs已被证实是肿瘤微环境的基质细胞中的关键细胞，这是因为它们在大多数实体肿瘤中含量丰富，并参与肿瘤的发生、发展及转移^[42]^。研究^[43-45]^表明，CAFs可以分泌大量VEGF-C以及其他的淋巴管生成因子，如HGF和PDGF，以诱导肿瘤相关的LECs增殖。Wang等^[46]^发现赖氨酰氧化酶样蛋白2（lysyl oxidase-like protein 2, LOXL2）可以刺激CAFs分泌大量促淋巴管生成因子VEGF-C和基质细胞衍生因子1α（stromal cell-derived factor 1α, SDF-1α），以促进淋巴管生成及肿瘤淋巴转移。与肿瘤细胞和LECs间相互作用相似，LECs也可以反过来作用于CAFs。Thomson等^[47]^证实了表达非典型趋化因子受体4（atypical chemokine receptors 4, ACKR4）的CAFs可以被LECs来源的CCL21所调控，并且通过免疫荧光染色发现这种CAFs在空间上也与淋巴管相邻，为两者间相互作用提供了空间上的可能性。

### 2.3 肿瘤相关免疫细胞与LECs的相互作用

在肿瘤的发展过程中，肿瘤相关免疫细胞可影响肿瘤微环境，调控肿瘤的生存、增殖和转移等生物学行为，其中包括巨噬细胞、T细胞、树突状细胞（dendritic cells, DCs）等^[48]^。巨噬细胞是肿瘤微环境中含量最丰富的免疫细胞^[49]^，可以通过分泌脂质运载蛋白2（lipocalin 2, LCN2）激活VEGF-C-VEGFR-3通路来促进淋巴管生成。同时，Watari等^[50]^研究发现NSCLC的肿瘤相关巨噬细胞（tumor-associated macrophages, TAMs）在受到IL-1β的刺激后，可以大量分泌VEGF-C/D以启动和维持淋巴管生成。另外，调节性T细胞（regulatory T cells, Tregs）可通过淋巴毒素（lymphotoxins, LTs）作用于LECs上的LTβ受体，从而诱导其产生趋化分子并促进LECs的跨内皮迁移^[51]^。相反的，LECs也可以作用于免疫细胞，调控其增殖、迁移等。例如淋巴结内LECs可表达一氧化氮合酶2（nitric oxide synthase 2, NOS2）以促进产生一氧化氮，抑制T细胞的增殖^[52]^。Moran等^[53]^的研究发现，LECs产生的生长因子和趋化因子是产生被膜下淋巴窦巨噬细胞的必要因素。此外，肿瘤相关LECs还可分泌CCL21与DCs表面表达的受体CCR7相结合，促进其进入淋巴系统，进而向淋巴结内的T细胞呈递抗原^[54]^。

### 2.4 肿瘤细胞外基质（extracellular matrix, ECM）对LECs的作用

肿瘤ECM是指在肿瘤微环境中为不同类型细胞提供生存环境并参与其生物学活动的非细胞成分^[55]^。ECM对肿瘤相关LECs也同样有支持作用和促进淋巴管生成的作用。一方面，ECM蛋白可以为LECs提供必要的结构支持，如胶原蛋白和纤维粘连蛋白等^[56, 57]^。此外，LECs表达的整联蛋白（integrins）可促进细胞与ECM的纤维粘连蛋白相粘附，这是LECs锚定的必要条件，同时还会使LECs的VEGFR-3磷酸化以促进淋巴管生成^[58, 59]^。另一方面，通过基质金属蛋白酶（matrix metalloproteinase, MMP）可以使ECM发生重塑，形成更疏松的环境进而促进淋巴管生成。例如，MMP-2作为一种胶原酶，可以驱动LECs在胶原基质中迁移^[60]^。此外，ECM能够产生透明质酸（hyaluronic acid, HA），然后与LECs的标志物LYVE-1相结合，促进其增殖和迁移并显著促进肿瘤内淋巴管生成^[61]^。

## 3 肿瘤相关淋巴管生成的调控机制研究进展

上述大量关于淋巴管生成的机制研究为肿瘤包括NSCLC提供了许多潜在的诊断标志物及治疗靶点。但是目前关于淋巴管生成的细胞内机制的研究仍旧比较缺乏。近年来，随着对非编码RNA的研究逐渐深入，这一类原先被认为是无调控功能的副产物引起了广大研究人员的重视，越来越多肿瘤相关淋巴管生成的分子机制得到了进一步阐明。

微小RNA（microRNA, miRNA）作为一种小非编码RNA，因其对基因表达的转录后调控而受到关注，miRNA可直接靶向mRNA以调控转录或破坏其稳定性来发挥作用。Hu等^[62]^发现miR-128的表达水平在NSCLC中呈低表达，并且还证实miR-128直接与VEGF-C的mRNA 3’-UTR结合以抑制VEGF-C表达。此外，在NSCLC细胞中过表达miR-128可以观察到VEGFR-3的表达下调。该项研究最终阐明miR-128在NSCLC中可以靶向VEGF-C-VEGFR-3通路以抑制淋巴管生成。miRNA还可以在其他多种肿瘤中调控淋巴管生成，例如miR-655和miR-526b在乳腺癌中对淋巴管生成呈促进作用^[63]^，miR-548k也通过VEGF-C-VEGFR-3通路在食管鳞癌中发挥正向调控^[64]^，而miR-27b对胃癌中的淋巴管生成却是呈抑制作用^[65]^。

长链非编码RNA（long non-coding RNA, lncRNA）是一类长度约200个核苷酸的非编码RNA。与miRNA不同，lncRNA可与RNA、DNA、蛋白质等分子相互作用，从而调控mRNA转录、基因表达和蛋白质功能等^[66]^。目前关于lncRNA调控淋巴管生成的研究主要集中于其他肿瘤，针对NSCLC的研究还比较缺乏。例如：Sun等^[67]^研究发现高表达lncRNA ANRIL的结直肠癌患者会出现VEGF-C、VEGFR-3和LYVE-1的高表达。进一步的研究表明，抑制ANRIL表达显著降低了LECs的侵袭、迁移和成管。综上所述，ANRIL可能作为结直肠癌淋巴管生成的驱动因素。除此之外，lncRNA BLACAT2也被证实可以通过与WDR5蛋白直接结合以调控VEGF-C，从而促进膀胱癌淋巴管生成^[68]^。

环状RNA（circular RNA, circRNA）代表一类新型非编码RNA，其具有共价环状结构，源自前体mRNA的非规范剪接，与线性对应序列的核酸相比其结构更加稳定^[69]^。circRNA通常定位于细胞质，这类circRNA大部分起miRNA海绵（miRNA sponges）的作用^[70]^。与lncRNA情况类似，目前尚缺乏circRNA在NSCLC的淋巴管生成方面的研究，但在膀胱癌及胰腺癌中已有相关报道。首先，Zhu等^[71]^报道了在膀胱癌中circEHBP1通过海绵吸附miR-130a-3p，以抑制该miRNA对TGFβR1的表达下调作用，而TGFβR1可使SMAD3发生磷酸化，因此circEHBP1可以间接增强SMAD3的磷酸化，进而促进VEGF-D依赖的淋巴管生成作用。此外，Kong等^[72]^也证实了circNFIB1也可通过吸附miR-486-5p以抑制其对PIK3R1的转录后调控，最终导致胰腺癌中VEGF-C依赖的淋巴管生成及淋巴转移受抑。

## 4 总结与展望

淋巴转移是NSCLC的主要转移途径之一，也是预测患者预后不良的重要因素，是否发生淋巴转移是对NSCLC患者进行临床分期以及进一步制定治疗策略的重要依据。淋巴管生成在促进肿瘤淋巴转移的过程中起至关重要的作用。因此无论是过去对于肿瘤细胞与LECs之间信号通路的揭示，还是近年来对于肿瘤微环境与LECs间相互作用的探索，甚至是目前细胞内非编码RNA对淋巴管生成的相关调控研究，都对于发现新的肿瘤标志物和治疗靶点有重要意义。然而目前相关研究主要集中于乳腺癌、黑色素瘤以及膀胱癌等癌种，针对NSCLC的研究相对缺乏，尤其是非编码RNA方向的研究。因此，需要不断深入对于NSCLC相关淋巴管生成的分子机制研究，以期抗淋巴管生成的靶向治疗会成为NSCLC治疗的新方向。
